# Biological links between traumatic brain injury and Parkinson’s disease

**DOI:** 10.1186/s40478-020-00924-7

**Published:** 2020-04-07

**Authors:** Vedad Delic, Kevin D. Beck, Kevin C. H. Pang, Bruce A. Citron

**Affiliations:** 1 Laboratory of Molecular Biology, VA New Jersey Health Care System, Research and Development (Mailstop 15), 385 Tremont Ave, East Orange, NJ 07018 USA; 2 NeuroBehavioral Research Laboratory, VA New Jersey Health Care System, Research and Development (Mailstop 15), 385 Tremont Ave, East Orange, NJ 07018 USA; 3 Department of Pharmacology, Physiology, and Neuroscience, Rutgers- New Jersey Medical School, Newark, NJ 07103 USA

## Abstract

Parkinson’s Disease (PD) is a progressive neurodegenerative disorder with no cure. Clinical presentation is characterized by postural instability, resting tremors, and gait problems that result from progressive loss of A9 dopaminergic neurons in the substantia nigra pars compacta. Traumatic brain injury (TBI) has been implicated as a risk factor for several neurodegenerative diseases, but the strongest evidence is linked to development of PD. Mild TBI (mTBI), is the most common and is defined by minimal, if any, loss of consciousness and the absence of significant observable damage to the brain tissue. mTBI is responsible for a 56% higher risk of developing PD in U.S. Veterans and the risk increases with severity of injury. While the mounting evidence from human studies suggests a link between TBI and PD, fundamental questions as to whether TBI nucleates PD pathology or accelerates PD pathology in vulnerable populations remains unanswered. Several promising lines of research point to inflammation, metabolic dysregulation, and protein accumulation as potential mechanisms through which TBI can initiate or accelerate PD. Amyloid precursor protein (APP), alpha synuclein (α-syn), hyper-phosphorylated Tau, and TAR DNA-binding protein 43 (TDP-43), are some of the most frequently reported proteins upregulated following a TBI and are also closely linked to PD. Recently, upregulation of Leucine Rich Repeat Kinase 2 (LRRK2), has been found in the brain of mice following a TBI. Subset of Rab proteins were identified as biological substrates of LRRK2, a protein also extensively linked to late onset PD. Inhibition of LRRK2 was found to be neuroprotective in PD and TBI models. The goal of this review is to survey current literature concerning the mechanistic overlap between TBI and PD with a particular focus on inflammation, metabolic dysregulation, and aforementioned proteins. This review will also cover the application of rodent TBI models to further our understanding of the relationship between TBI and PD.

## Introduction

TBI has been implicated as a risk factor for several neurodegenerative diseases, including Alzheimer’s disease (AD), Amyotrophic Lateral Sclerosis (ALS), and PD [148]. Compelling evidence from three prospective cohort studies aimed at determining the relationship between TBI, AD, and PD supports the relationship between TBI and PD pathology [8, 9, 30, 76]. Analysis of aggregate neuropathological data gathered from Adult Changes in Thought study, the Religious Orders Study, and the Memory and Aging Project, all prospective studies, indicate that TBI is associated with a risk for Lewy body accumulation, parkinsonism, and PD and that TBI is not associated with dementia and AD pathology [30]. Furthermore, a recent study has shown that Veterans with a history of mTBI are at a 56% higher risk for developing PD later in life and that this risk grows with increased TBI severity [45]. According to the CDC, an estimated 1.7 million people sustain a TBI annually in the U.S., and approximately 20% of returning service personnel are affected by TBI, totaling 320,000 soldiers since 2000 [106]. The high prevalence of TBI and its role as a risk factor for PD is a significant cause for concern.

Men account for the majority of TBIs in the U.S. and are also twice as likely to be diagnosed with PD [108, 151]. Although the sex differences are not fully understood, some studies suggest that estrogen could be responsible for the neuroprotection and milder symptoms seen at the onset of PD developing in women [19, 49]. Other studies report that the progression of PD after onset is more aggressive in women than men which calls the estrogen explanation into question [10, 26, 130]. While the influence of sex on PD remains under investigation, less is known about the mechanistic contributions of TBI that could lead to eventual PD pathology.

In the general U.S. population, diagnosis of PD in individuals over the age of 45 is estimated to reach 930,000 by 2020 and 1,238,000 by 2030 [98], forecasting a significant long-term disability cost to patients and their communities. Treatments beyond palliative care are not yet available for either PD or long-term consequences of TBI nor are there interventions known to slow down or prevent PD in at risk populations. Understanding the mechanistic relationship between PD and TBI will help inform future studies toward effective treatments and preventions. The following sections will contextualize disease overlap between PD and the neuropathology observed following TBI, highlight the most common types of TBI models and their applications in PD research, and discuss the next steps necessary to help determine points of intervention that may prevent PD from developing after TBI.

### Pathology overlap between TBI and PD

TBI sequalae can be divided in to 3 phases: acute (0 to 1 week), post-acute (1 week to 1 month), and chronic (1 month to years). During the acute period cell necrosis from direct transfer of force to the brain tissue occurs, followed by secondary cell death from axonal pathology, and inflammation. The post-acute period can be characterized by neuronal remodeling, decreased inflammation, and an increase in chronic pathology [89]. Chronic pathology of most interest here includes, neurodegeneration, protein misfolding (α-syn, APP, Tau, TDP43), and persistent inflammation. Chronic TBI pathology can vary, with some patients recovering completely while others suffer physical and cognitive decline, and eventually develop neurodegenerative diseases [146], most notably Parkinson’s Disease (PD) [30].

PD is the most prevalent movement disorder for which TBI is a major non-genetic risk factor [30, 61]. PD affects the entire basal ganglia, and is classically characterized by dopamine dysregulation in the nigrostriatal circuit, neuronal mitochondrial dysfunction, loss of dopaminergic projections to the striatum from the Substantia Nigra pars compacta (SNpc), culminating in progressive death of A9 dopaminergic neurons (DNs). A9 DNs of the SNpc are selectively lost in PD, while adjacent A10 DNs in the ventral tegmental area (VTA) are spared. This interesting selective loss of A9 neurons in PD is not yet fully understood, but it could be attributed to the A9 DN size, and higher expression of mitochondrial and metabolic proteins compared to other DNs [24]. Although motor symptoms remain cardinal signs associated with clinical PD, more recent clinical findings show that early PD pathology can cause non-motor symptoms that significantly impact life quality of patients.

Excessive salivation, constipation, forgetfulness, hyposmia and urinary urgency are common non-motor symptoms present in early PD that worsen with progression of the disease [71]. The most common psychotic phenomenon associated with PD are minor visual hallucinations [81]. Fourty to fifty percent of PD patients also develop anxiety which can occur at any stages of the disease [80, 120]. Emerging evidence suggests that PD related pathology may occur in the peripheral nervous system (PNS), but due to lack of drug-naïve groups it is difficult to distinguish if the PNS neuropathology is a consequence of PD or pharmacological PD treatments [27]. In light of wide-ranging non-motor symptoms, and presence of pathology outside of the CNS, PD is increasingly considered a systemic rather than a disease of basal ganglia. Non-motor symptoms and pathology in the PNS, therefore, deserve additional consideration when studying PD pathology spread in humans and in animal models and during development of PD therapeutics.

PD during life is well defined by a combination of motor and non-motor symptoms, but the only definitive diagnosis remains by autopsy. Presence of intracellular proteinaceous Lewy Body inclusions and thread-like Lewy neurites are required for definitive postmortem diagnosis of PD. Originally, Lewy Body and Lewy neurite pathology was visualized using antibodies raised against ubiquitin, but subsequent study by Spillantini et al (1998) demonstrated that the PD inclusions were made up predominantly of misfolded and aggregated α-syn protein [143]. Distinguishing posttranslational modification of the normally soluble and abundant α-syn is hyper phosphorylation at the serine 129 residue [43]. Within neurons, α-syn is normally localized to the synapse and the nucleus but the exact function of α-syn is not yet known. These inclusions do not spread indiscriminately and are found in susceptible neurons that share some common traits. Neurons that appear to be protected are extensively myelinated projection neurons with long or short axons and those that are vulnerable are incompletely myelinated metabolically demanding projection neurons with long and thin axons [14]. It would be of interest to determine if the same neuron types are made more susceptible to PD pathology following diffuse axonal injury caused by mild or repetitive TBI.

Cause of α-syn pathology nucleation remains unknown, but Braak hypothesized that an as of yet unknown pathogen could gain access to the central nervous system through the mucosa and neurons of the gut and the nasal cavity and nucleate PD pathology in those areas [51, 52]. According to the Braak hypothesis, a pathogen can gain entry into the CNS in one of two ways. One way is through the gut via the vagus nerve where the pathogen can nucleate α-syn pathology that then spreads up the brain stem to the SNpc and the other is through the nasal mucosa via the olfactory neurons [14]. From these locations, the pathology can spread sequentially over time through vulnerable neurons. Analysis of postmortem idiopathic PD brains by Braak and Braak (2003) lead to the development of PD pathology staging that continues to be widely used by physicians and scientists [13]. The staging assumes that PD pathology does not originate simultaneously in all the affected brain areas, that the pathology burden is lower early in the disease and progresses over time through interconnected susceptible neurons. The 6 Braak stages are organized based on lesion location and inclusion pathology, with stages 1-2 having the lowest PD pathology burden with inclusions confined to the medulla oblongota and stages 5-6 having the highest pathology burden with significant brain involvement including the neocortical areas [13]. Braak staging has been confirmed with a study reporting 12 percent of clinical PD cases classified as Braak stage 3 and 88 percent of clinical PD cases as having Braak stages 4-6 [62]. α-syn pathology was also found in the spinal and peripheral autonomic nervous system of 98 elderly patients without PD symptoms, suggesting that the earliest PD pathology may occur in the periphery and the spinal cord [12]. Braak hypothesis and staging however are not without their criticism. Because Braak hypothesis and Braak staging are based on the study of postmortem tissue from patients with young onset and long duration of the disease, Braak hypothesis and staging may only reflecta subset of PD cases [127].

Further evidence supporting the importance of α-syn in pathogenesis of PD is provided by rare familial mutations within the alpha synuclein gene (SNCA) that result in aggressive early onset PD [72, 75, 82, 118, 167]. In addition to the missense mutations, duplications and triplications in an otherwise normal SNCA gene can also cause PD [21, 57, 85]. Post mortem analysis of PD patient brains who underwent bilateral fetal mesencephalic tissue transplantation, showed phospho-serine 129 α-syn positive Lewy Body inclusions in the grafted tissue, indicating trans-neuronal spread from recipient tissue to naïve donor tissue [83]. These findings also implicate abnormal α-syn as a key player in the pathogenesis and pathology of PD. Mutations in other genes are also known to contribute to the development of PD with Lewy Body pathology. These include mutations in PARK2 (Parkin), PTEN-induced putative kinase 1 (PINK1), DJ-1, ATP13A2, and leucine-rich repeat kinase 2 (LRRK2) [32, 74].

**Figure 1:**
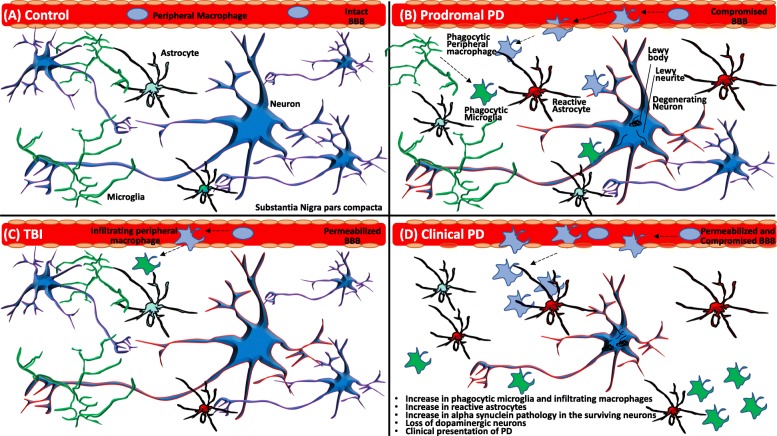
Potential influences of TBI on the course of PD. In the healthy brain the blood brain barrier (BBB) is intact, microglia are highly ramified and astrocytes are non-reactive and provide nourishment to neurons (**a**). In prodromal PD, degenerating neurons develop alpha synuclein pathology in the form of Lewy bodies and Lewy neurites, microglia become more amoeboid and phagocytic, BBB is compromised and peripheral immune cells infiltrate the brain where they become activated and take on a role similar to that of phagocytic resident microglia (**b**). After a TBI, BBB is temporarily permeabilized allowing infiltration of the peripheral immune cells, astrocytes become reactive and some neurons degenerate (**c**). In the clinical PD, the substantia nigra pars compacta is almost entirely devoid of dopaminergic neurons, and surviving neurons in nigra and other affected brain areas are burdened with alpha synuclein pathology while the areas vacated by dying neurons are filled with reactive astrocytes, microglia, and peripheral immune cells (**d**). Overlap in pathology induced by TBI and present in prodromal PD may allow for TBI to accelerate prodromal PD to clinical PD.

PD as well as TBI brains are characterized by neuronal degeneration, compromised blood brain barrier, infiltration and expansion of resident microglia into the affected areas, and infiltration of phagocytic cells from the periphery (Fig. 1). In both PD and following a TBI this histological presentation is accompanied by inflammation, metabolic disturbances, and protein aggregation, making them essential factors to consider when studying the mechanisms connecting these two disorders.

#### Inflammation

Markers of inflammation are present in postmortem human brains years after TBI and are also present in brains of PD patients [123, 159]. Brains examined by positron emission tomography (PET) following head trauma for an activated microglial marker, showed an increase in activated microglia up to 17 years after a TBI, which was found to be associated with impaired information processing [123]. Inflammation occurs shortly after initial TBI event (acute sequalae), and can persist for years (chronic sequalae) in the affected areas [134]. Therefore, inflammation can be seen as a “chronic response to an acute event.”

Similarly, persistent inflammation was found in PD patients with increased reactive microglia in the SNpc [101]. Major Histocompatibility Complex II (MHCII), normally not expressed in the brain, has been found in postmortem brains of PD patients [101]. MHCII is an important regulator of cellular innate immunity which also presents antigens to T and B lymphocytes of the adaptive immune system. CD8+ “killer” T cells are cytotoxic and can kill other infected or damaged cells. CD8+ T cells can recruit additional immune cells through the release of cytokines. B lymphocytes activated by MHCII and T cells, differentiate into memory B cells that retain information on the antigen, and undergo further maturation and expansion into antibody producing plasma cells. In the early stages of PD, MHCII expression correlates with toxic intracellular α-syn aggregation in neurons, suggesting that prior to cell death α-syn aggregation may be an important mediator of the immune response [58]. The number of activated microglia increases in the subtantia nigra with progression of PD and activated microglia are also found in other affected brain areas [132]. Unfortunately, detailed study of human PD and TBI brain inflammation remain limited to postmortem tissue necessitating reliance on animal models.

Templated prion-like conformational change of α-syn into oligomers and higher ordered structures is supported by abundant In vitro and in vivo evidence [93]. Most compelling evidence for the α-syn prion-like hypothesis comes from primate and rodent studies where primate or rodent brains were injected with misfolded α-syn from different sources and subsequently developed PD-like pathology. In these studies, α-syn PD-like pathology was induced by injection of human PD patient brain homogenate [124, 125], brain homogenate from transgenic animals that developed α-syn pathology [110], or injection of transgenic and non-transgenic animals with recombinant α-syn fibrils [92, 116, 129, 139]. These studies provide a direct line of evidence for α-syn pathogenic templating from human PD patient brain homogenate containing misfolded α-syn to transgenic animal models that demonstrate oligomeric α-syn toxicity, and finally a synthetic α-syn fibrils that can nucleate PD pathology. Together, these human and animal studies have paved the way for more controlled interrogation of α-syn driven PD pathology. For additional information on α-syn fibril and other PD models we direct the reader to a review by Volpicelli-Daley et al [157].

Using synthetic fibrilized α-syn, MHCII expression in rats, like in humans, has been shown to persist after dopaminergic neurons in the SNpc have degenerated and died [50]. In the same model, immune cells from the periphery were also recruited to the site of injury prior to neurodegeneration and MHCII expression was found at later time points in the striatum, as the inclusion pathology spread across the synapse from dopaminergic neurons in the SNpc to medium spiny neurons of the striatum [50]. Similarly, following a TBI in humans, activated microglia expand throughout the injured brain regions [114, 155]. In mice following a single controlled cortical impact, activated microglia and macrophages continue to express elevated levels of MHCII and CD68, a year after the injury [90].

Due to this similarity in immune response between PD and TBI, it is possible that TBI may accelerate underlying PD pathology in additive or synergistic manner thereby driving the subclinical PD pathology to overt PD pathology. Since both mice and rats exhibit similar immune responses as seen in human postmortem brain tissue, they are an adequate platform for further research that will help us understand how inflammation may drive TBI pathology toward development of PD.

#### Metabolism

The human brain consumes 20% of our calories but only accounts for 2% of the total body’s mass [40, 128]. Most of this energy is used by neurons for saltatory signal transduction, while the rest is expended on housekeeping processes which include responses to injury [40]. Astrocytes, endothelial cells, pericytes and supplying blood vessels form a physiological neurovascular unit that provides the neuron with nutrients and also serves as a buffer for minor fluctuations in energy supply. Following a TBI, neurovascular unit damage results in disruption of homeostasis, excitotoxicity, production of reactive oxygen species, and inflammation [44, 66]. Prolonged disruption of energy homeostasis, excitotoxicity, and damage from reactive oxygen species (ROS) promotes neuroinflammatory signaling cascade [15, 66, 163]. These events continue beyond the initial injury which may present an opportunity for therapeutic intervention, but may also create an environment conducive to nucleation of PD.

Nucleation of PD may be initially precipitated by decreased supply of metabolic nutrients and oxygen caused by damage to the blood brain barrier (BBB). Disruption of controlled and highly selective transport across the BBB results in a release of cytosolic and mitochondrial Ca^2+^, triggering intracellular catabolic processes accompanied by overproduction of ROS in neurons. Most of the neuronal ATP is produced through oxidative phosphorylation from densely packed neuronal mitochondria. Disruption of a delicate network of interconnected mitochondria undergoing near constant fusion and fission can result in inadequate energy production, decreased neuronal mitochondria membrane potential and increased production of ROS [64].

Intact mitochondrial membrane and functional electron transport chain complexes are essential for maintaining membrane potential between mitochondrial matrix and the cytosol [100]. This membrane potential is converted to usable energy by coupling of oxygen reduction to phosphorylation of ADP to ATP [100]. Damaged electron transport chain or compromised inner mitochondrial membrane resulting from TBI can cause increased ROS production along with decrease of ATP in neurons.

4-hydroxy-2-nonenal, a biproduct of membrane lipid peroxidation by ROS, can interact with soluble and highly abundant α-syn protein and promote its aggregation into toxic oligomers in a dose dependent manner [4, 122]. Diverse lines of evidence show that α-syn oligomers can interact with and disrupt mitochondrial function by impeding axonal transport of mitochondria [121], causing mitochondrial membrane destabilization and permeability [47, 160] and by interrupting electron transport chain machinery [91]. These are all components essential for ATP production. Inadequate energy production can exacerbate oligomerization of α-syn by slowing down the energy-demanding autophagy-lysosomal and ubiquitin-proteasome degradation pathways. Both degradation pathways are involved in α-syn turnover [161]. Over time, toxic oligomers could overwhelm the mitochondria’s ability to maintain homeostasis, resulting in the death of energy demanding neurons like A9 dopaminergic neurons. As a last resort, degenerating neurons may sequester toxic α-syn oligomers into the higher ordered cytosolic fibrils and Lewy bodies that are a hallmark of PD pathology.

Repeated brain injuries over the years may accelerate existing pathology resulting in earlier age of onset and increased severity within the late onset PD population. Perhaps repeated injuries in adulthood, may be more important at accelerating underlying PD pathology than those that occur in childhood, since younger brains recover more effectively than older brains after a TBI [97]. Additional support for the idea that TBI in adulthood may be more likely to nucleate or accelerate PD pathology comes from work by Uryu et al 2003. In their study, 24 month old mice that underwent controlled cortical impact TBI show more extensive, albeit transient conformational and nitrated α-syn pathology compared to mice injured at 4 months [153].

#### Protein aggregation

Protein aggregation is a feature of many neurodegenerative diseases and is also a consequence of repeated head injury, as seen in the pathology of chronic traumatic encephalopathy (CTE) [104]. Further insight into the proteins most frequently found to aggregate both in PD and following TBI may shed light on as of yet elusive molecular connections.

*α-syn* is an intrinsically disordered protein whose amyloidogenic properties and involvement in PD are well documented. α-syn is made up of 140 amino acids encoded by the SNCA gene on chromosome 4q21-q23 [22]. Under physiological conditions, most of the α-syn in the brain is not phosphorylated, whereas in brains of patients with PD and other synucleinopathies Lewy bodies are almost exclusively made of α-syn hyper-phosphorylated at serine 129 residue [115]. α-syn is highly abundant in neurons, and is concentrated in the nucleus and the synapse. While the precise biological function of α-syn remains a mystery, its affinity toward certain phospholipid membranes and localization at the synapse has led to its implication in vesicular trafficking [7]. The exact molecular events that trigger the conversion of intrinsically disordered α-syn into the pathologic oligomers and later sequestration into higher ordered structures seen in neurodegenerative diseases is also unclear. However, what is known is that α-syn conversion into the pathogenic forms can be accelerated in vitro in a dose dependent manner with byproducts of free radical lipid peroxidation (e.g. 4-hydroxy-2-nonenal) and higher concentration of α-syn [4, 154]. Pesticides and heavy metal exposure has been linked to development of PD [126] and their addition in vitro has also been shown to accelerate oligomerization and aggregation of α-syn [154]. In fact, fractioning fibrilized α-syn by sonication into smaller fragments and injecting that material into the brains of rodents causes human like PD pathology [1]. This is a model of PD that at least in terms of α-syn aggregate morphology most closely resembles pathology seen in idiopathic PD [157].

In vivo, the connection between PD pathogenesis and suspected risk factors is less clearly defined. α-syn is elevated in the CSF of patients with TBI and also increases proportional to the severity of injury [109, 147]. In these studies, adults and children with a Glasgow Coma Score < 8.0 received a ventricular catheterization to allow for continuous drainage in an effort to alleviate intracranial pressure during which the CSF samples were collected. Patients without TBI that underwent a lumbar puncture for other medical reasons were used as controls. In both children and adults, higher levels of α-syn in the CSF were associated with higher severity of injury [109, 147]. In rodent controlled cortical impact-TBI models, α-syn is elevated and is accompanied by neurodegeneration but without robust serine 129 positive Lewy body pathology that defines PD [2]. Following a TBI, elevated α-syn, increased ROS (promoting oligomerization), and compromised proteasomal function together may create an environment that promotes early PD pathology. From previous studies we know that free radical lipid peroxidation (e.g. 4-hydroxy-2-nonenal) and higher concentration of α-syn can promote oligomerization into more toxic α-syn aggregates [4, 154]. It is therefore possible that higher concentration of α-syn that accompanies TBI, together with damaged mitochondria which produce more ROS, may foster an environment conducive to PD pathology nucleation.

*Amyloid beta* (Aβ) is an approximately 4 kDa, 36-43 amino acid peptide, produced by cleavage of amyloid precursor protein (APP). The exact function of APP is not yet known but the production of APP and its processing is now well understood [112]. APP undergoes sequential proteolytic cleavage by two membrane bound endoproteases β- and a γ-secretase. First, APP is cleaved by β-secretase releasing a large fragment sAPPβ. The remaining membrane bound 99 amino acid fragments are cleaved by γ-secretase generating Aβ. Along with Tau, which is discussed later in the text, Aβ is most commonly associated with Alzheimer’s disease (AD) when it aggregates into higher order parenchymal “senile plaques”. While Aβ plaques were thought to be the causative agent of neuronal degeneration in AD, more recently oligomeric precursors have been shown to have even higher toxicity [137]. Like α-syn, TDP-43, and Tau, misfolded Aβ can “corrupt” soluble Aβ and propagate aggregation in a prion-like fashion [136]. Unlike α-syn, aggregation of Aβ can be intracellular and extracellular, causing death of a variety of neurons in hippocampus, frontal cortex, and subcortical nuclei [138] . Studies show that Aβ toxicity can be receptor-mediated, occur by destabilizing plasma membranes, and by intracellular toxicity [70]. Extracellular Aβ can interact with N-methyl-D-aspartate receptor (NMDAR) leading to synaptic dysfunction followed by neurodegeneration leading to functional disruption [142]. Intercellular Aβ is localized in mitochondrial membrane, endoplasmic reticulum, and lysosomal membranes where cytotoxicity is achieved by disruption of proper organelle function [70].

Levels of Aβ fragment and senile plaques increases after a single TBI, when compared to age matched controls [65]. Postmortem brain tissue 1-47 years after a single TBI have Aβ plaques throughout the brain while the plaques were exceedingly rare in age matched controls. These data suggest that just a single TBI event can predispose the brain to AD or PD later in life. In rodents even mild repetitive TBI has been shown to promote Aβ pathology [38].

In a subpopulation of patients with Lewy body spectrum disorders, deposition of Aβ was found along with the Lewy bodies in dementia with Lewy bodies and to a lesser extent in PD dementia patients [37]. Over 80% of PD patients surviving more than a decade will eventually develop dementia, although the severity and onset can vary by years and sometimes decades [36]. In transgenic animals and in vitro, studies Aβ was found to accelerate α-syn aggregation into Lewy bodies and that α-syn can in turn accelerate Aβ aggregation [25, 28, 77, 99, 117]. Understanding the interaction between AD and PD pathology can help inform the search for treatments. Moreover, drugs found to be effective in human trials for AD [135] may be repurposed for treating PD, particularly at staving off dementia, since motor disturbances precede PD mental decline and dementia by years and sometimes decades.

*TAR DNA-binding protein 43* (TDP-43) is most notably associated with amyotrophic lateral sclerosis (ALS), and over 50 mutations in the TARDP gene coding for TDP-43 have been found to cause ALS [111]. TDP-43 is normally located in the nucleus of most cells, and thousands of genes are transcriptionally regulated by TDP-43. In the pathology of neurodegenerative disorders including chronic traumatic encephalopathy (CTE) and frontotemporal lobar degeneration, TDP-43 positive inclusions are found in the cytoplasm [6, 105]. TDP-43 positive inclusions also occur in patients with diagnosed PD and other neurodegenerative disorders with α-syn pathology such as dementia with Lewy bodies [113]. TDP-43 is cleaved into 25 and 35 kDa fragments, which are hyperphosphorylated, ubiquinated and aggregated into these inclusions. While limited clinical research exists on the relationship between chronic TBI and TDP-43, behavioral symptoms normally associated with frontotemporal dementia (FTD) like apathy and social dysfunction also frequently occur in patients with TBI and CTE [150]. These symptoms are thought to occur as a result of frontal lobe degeneration.

Various animal models of TBI have been used extensively to interrogate TDP-43 proteinopathy [54]. Using either repetitive controlled cortical impact (CCI) or fluid percussion injury (FPI) TDP-43 fragments are found to be mislocalized to the cytoplasm where they are phosphorylated, causing neuronal death and impaired cognition in memory tests [149, 158, 164]. TDP-43 proteinopathy was also achieved in rodents using less severe repetitive closed head blast injury models. Exposure of anesthetized mice to 30 psi overpressure blast wave resulted in TDP43 proteinopathy, compared to controls which were only exposed to the blast sound and not the blast wave [166]. In a similar injury model, rats were exposed up to 4 repetitive mild blasts consisting of 13, 16, or 19 psi injuries delivered to the front or the side of the head resulting in TDP-43 proteinopathy [53]. Although TDP-43 positive inclusions occur in patients with α-syn pathology, we found no evidence in literature of TDP-43 interaction or colocalization with Lewy bodies or Lewy neurites in humans. Future studies focused on determining chronic outcomes of TBIs that produced TDP-43 pathology in PD animal models with robust α-syn pathology are necessary to determine how TDP-43 pathology fits in mechanistically with PD.

*Tau* is a highly soluble member of the microtubule-associated protein (MAP) family, responsible for stabilizing microtubule assembly networks. Neuronal Tau include MAP-2A and MAP-4 (> 200 kDa), as well as a smaller MAP-2C ( > 80 kDa ) [95]. Like α-syn and Aβ, Tau has a well-established role in pathology of AD, PD and TBI. Of the two haplotypes H1 and H2, H1 has the strongest association with PD. The H1 haplotype, present in 87% of PD cases and 77% of controls is associated with increased risk for PD compared to the H2 haplotype [152]. Pathological presentation of Tau in neurodegenerative diseases like PD, and as seen in TBI, is in the form of neurofibrillary tangles (NFTs) resulting from Tau hyper phosphorylation. Hyperphosphorylation of Tau causes both loss of function leading to destabilization of the microtubule network and deposition of toxic aggregates, which can in turn affect structure and transport throughout the cell [79]. In idiopathic PD, Tau and α-syn are colocalized in Lewy bodies [60]. Tau-positive Lewy bodies can be classified into four types based on immunoreactive Tau localization within the aggregates [60]. While widespread aggregation of α-syn and Tau occurs in Lewy bodies in postmortem tissue from patients with various synucleiopathies (e.g. Dementia with Lewy bodies, Parkinson’s disease, Frontotemporal dementia) the localization does not follow any particular Braak staging of disease severity [60]. Further evidence for a biological relationship between α-syn and Tau is provided by in vitro and animal model studies.

In vitro protein interaction studies using bindings assays have shown that the C-terminus of α-syn domain binds reversibly to the microtubule binding domain of Tau and that this binding stimulates phosphorylation of Tau at serine residues 262 and 356 [63]. Transgenic mice harboring the rare familial A30P α-syn mutation show increased Tau phosphorylation at serine residues 396 and 404 [42]. Western blot analysis demonstrates that immunoreactive Tau and α-syn are significantly higher in A30P symptomatic mice than in A30P asymptomatic mice or age-matched non-transgenic littermate controls [42]. These studies together provide compelling evidence for a direct pathological relationship between Tau and α-syn proteins in PD.

Tau has also been implicated in TBI pathology. High-profile cases of athletes with CTE have garnered a great deal of media attention. CTE prevalence is still being elucidated along with additional details for diagnostic criteria [145]. CTE is a term used to describe brain degeneration that is likely caused by repetitive TBIs. As with most neurodegenerative disorders, confirmation is only possible postmortem, where Tau pathology is deposited within the cortical sulci around small blood vessels [46]. While TDP-43 is found in majority of CTE cases and amyloid beta pathology is found in 43% of CTE cases, the presence of α-syn pathology like Lewy bodies, is less clear [107]. Conclusive diagnosis of CTE is made more difficult because a significant portion of those diagnosed with CTE also present with pathology of other neurodegenerative diseases. Mckee et al. (2015) have shown that in 142 confirmed cases of CTE, 37 percent of patients shared comorbid pathology with one or more other neurodegenerative disorders including Alzheimer’s Disease and Lewy body disease [107]. Other studies have shown that although Lewy Body pathology can occur in CTE, clinical presentation of Parkinsonism is not yet associated with CTE, and, to date, severity and frequency of injury necessary to cause Lewy body pathology is not known [3]. Future animal studies focused on Lewy Body pathology and TBI are warranted to provide much needed clarity on the severity and frequency of TBI necessary to cause CTE and/or PD related α-syn pathology.

### Models of TBI and their application in PD research

While no single animal model of TBI will ever recapitulate all features of human TBI, each of the models discussed below can answer specific questions about aspects of human TBI.

Certain mechanistic questions can be best addressed with primary or model neurons in culture. One such method involves growing neuronal cells on a silastic membrane that is then stretched using compressed gas mimicking torsional stress experience by neurons during TBI. This biaxial stretch injury model has been adopted for use in a variety of immortalized and primary cells and has led to a better understanding of primary astrocyte and immortalize neuron cell specific responses to injury [39, 68]. In both studies, cell injury controllers were used to deliver a stretch injury to a monoculture of cells on a silastic membrane. The in vitro studies show that cellular responses are quantifiable and track with severity of injury in both astrocytes and immortalized neuronal cells.

To model TBI in vivo, several injury systems have been developed that cause skull penetrating and non-penetrating injuries in cats, dogs, ferrets, mice, monkeys ,rats, and swine [165]. Rodent TBI models are the most extensively used and are summarized in (Table 1). It is important to note that most injury models involve preparatory surgeries (i.e. a craniotomy or skull exposure). Surgery prior to the intended TBI injury is an important consideration because it may prime the animal’s immune system, thereby unintentionally enhancing the injury-specific inflammatory response. Modeling repetitive injuries, especially those separated by a significant time interval, may not be possible in TBI models that require surgeries. While repetitive closed head weight drop injuries that do not require preparatory surgeries have been well-documented in mice [133], they remain largely uncharacterized in rats. A modified Marmarou injury system developed by Buchele et al (2016) is a good candidate for further development into a repetitive TBI rat model without an incision. Additional consideration for modeling TBI in animals is the use of analgesia that is also anti-inflammatory. Aforementioned emerging evidence discussed above shows that inflammation in the form of infiltrating phagocytic cells from the periphery may help drive PD pathology. Therefore, the use of anti-inflammatory analgesics during or after injury may confound the results of the study and how it may relate to PD pathology. Research specifically focused on determining a biological relationship between TBI and PD has not been extensive and warrants increased efforts.

**Table 1:** Rodent TBI injury models. Controlled cortical impact (CCI), Fluid percussion injury (FPI), Penetrating ballistic brain injury (PBBI), and closed head injury (CHI) weight drop are the most commonly used injury methods

Model	Injury type	Preparatory surgery	Rodent
**Blast**	Diffuse	None	Mouse [48], Rat [69]
**CCI**	Focal	Craniotomy	Mouse [141], Rat [35]
**FPI**			
Middle	Mixed	Craniotomy	Rat [102]
Lateral	Mixed	Craniotomy	Mouse [17], Rat [103]
Repetitive	Diffuse	Craniotomy	Rat [34]
**PBBI**	Focal	Cranial incision, burr hole	Rat [162]
**Weight drop**			
Buchele	Diffuse	Cranial incision	Rat [16]
Citron	Repetitive, diffuse	None	Mouse [133]
Marmarou	Diffuse	Cranial incision, reflected periosteum	Rat [96]
Maryland model	Diffuse	Infraorbital incisions, temporary implant	Rat [73]
Pick	Diffuse	None	Mouse [169]
Shohami	Focal	Cranial incision	Mouse [23], Rat [140]

#### Blast TBI

Exposure to a blast wave from detonation of friendly and enemy explosive ordinances is a signature injury of recent conflicts. Following a detonation, the surrounding air is displaced by rapidly expanding gases at high velocity. This rapid blast wave is followed by blast wind which can reach hurricane level forces and causes dismemberment and lacerations [59]. Therefore, a person exposed to an explosion suffers two injuries in rapid succession. Severity of exposure to the blast wave depends on the magnitude of the positive pressure wave where an overpressure of 414-552 kPa or 60-80 psi can be potentially lethal [59]. Another important consideration even at a distance from the explosion, is the reflection of a blast wave from nearby objects and buildings. A growing body of research exists on how blast injuries affect the brain and other organs in mice and rats, using blast tubes that are more reproducible and more readily accessible than explosive ordinance detonation [94]. However, only a handful of studies exist on how blast injuries affect the progression of Alzheimer’s disease, and none exist on how blast injuries affect the progression of PD. Considering that blast injury is the signature type of TBI in recent conflicts and that Veterans with history of TBI are at a 56% higher risk for developing PD [45], blast in the context of PD pathology deserves additional research efforts.

#### Controlled cortical impact (CCI) TBI

CCI consists of an impactor that delivers an injury to the exposed brain and has been extensively used in TBI research with over 2,600 publications to date. A typical CCI device is made up of an inert gas driven piston that directly impacts the brain resulting in controlled brain deformation. Using a pneumatic impactor, Dixon et al. (1995) performed injuries on anesthetized SD rats with 3 injury levels [35]. The CCI device was built at the Medical College of Virginia and was made up of a pneumatic cylinder with an adjustable impact velocity [35]. Dixon et al. performed acute and chronic neurological assessments and cardiovascular responses and also evaluated the histopathology of the injured animals. Acute neurological assessments consisted of non-postural somatosensory functions by recording duration of reflex suppression to noxious stimuli. Postural somatosensory function was evaluated by measuring duration of suppression of the righting reflex and escape. Chronic neurological deficits were measured by latency to cross a beam-balance. Cardiovascular response to injury was determined by measuring arterial blood pressure, and histopathology was evaluated by noting parenchymal bleeding and axonal swelling visualized using antibodies targeted at all three neurofilament subunits. In general, the evaluations tracked with severity of injury. Both acute and chronic evaluations exhibited graded response to the severity of injury with suppressed responses to noxious stimuli, increased beam balance time, and increased latency to cross. Animals that sustain low injury do not show damage beyond the immediate point of contact with the impactor, while moderate to high injury result in bilateral intraparenchymal bleeding. Staining with antibodies for neurofilament subunits shows axonal swelling characterized by “retraction balls” present throughout the brain. A similar study was performed in C57BL mice by Smith et al. (1995), showing approximately 50% memory deficits using the Morris Water Maze and extensive gliosis, indicated by staining for a glial fibrillary acidic protein (GFAP), a marker of astrocytes [141]. Foundational studies by Dixon and Smith have guided the TBI field for decades, as evidenced by over 1,500 citations.

CCI provides focal and highly reproducible injuries, resulting in improved animal studies. However, the open skull, cortical deforming nature of the injury is seldom seen in human TBIs. The major limitations of this model are that it requires a craniotomy and that even mild injuries result in observable cortical deformation. Moreover, this model does not lend itself to repetitive mild injuries which may drive the pathology of neurodegenerative diseases in humans. In PD studies there has been limited application of CCI, totaling in only three studies that evaluated functional and histological deficits closely associated with PD. Acosta et al. (2015) found that 60 days after a CCI, there is an approximately 25% reduction in dopaminergic tyrosine hydroxylase-positive neurons in the ipsilateral SNpc and a two-fold increase in expression of α-syn in the surviving neurons [2]. Loss of dopaminergic neurons in the SNpc was accompanied by an approximately 20-fold increase in the volume of MHCII expressing microglia, in the SNpc [2]. However, no phospho-Serine 129-positive Lewy body-like inclusion pathology was reported. Leucine rich repeat kinase 2 (LRRK2) is a well-established contributor to both late onset familial and idiopathic PD. Studies show that gain of function mutations in LRRK2 increase susceptibility to α-syn pathology and that LRRK2 inhibition or knockout provides neuroprotection in multiple PD models [31, 86, 156]. More recently, LRRK2 expression was found to increase in CCI mouse TBI model, and LRRK2 inhibition was found to be neuroprotective [5]. Bae et al. (2018), show that inhibition of LRRK2 provides robust neuroprotection after a CCI in the cortex and hippocampus and that this neuroprotection results in improved motor function (beam balance) and memory (novel object recognition) tests.

Exposure to neurotoxins has been shown to cause PD later in life [126]. Exposure to neurotoxins may increase susceptibility to subsequent injuries like TBI that bring about clinical presentation of PD. Trichloroethylene (TCE), a halogenated hydrocarbon used as a solvent in the U.S., has been shown to cause PD-related functional deficits that are further exacerbated by TBI [131]. Using a CCI model, Sauerbeck et al. (2012) showed that exposure to either TCE or CCI alone does not result in dopaminergic neuron loss in the SNpc, but exposure to both causes 13-17% loss, suggesting a synergistic relationship between TBI and TCE exposure [131]. They also show mitochondrial dysfunction in the striatum without the loss of dopaminergic innervation from the SNpc. Although these three studies indicate a strong relationship between TBI and PD, key mechanistic questions remain unanswered, such as the relationship between TBI and trans-synaptic spread of more authentic α-syn Lewy body-like pathology consisting of dense perinuclear aggregates or corkscrew Lewy neurite-like structures as seen in human idiopathic PD [143]. Studies aimed at determining how focal injuries using CCI affect existing α-syn pathology would greatly improve our knowledge on the dynamics and mechanisms between TBI to specific brain areas and development of PD related pathology.

#### Fluid percussion injury (FPI)

Fluid percussion Injuries continue to be used in animal studies since first described in 1966 by Lindgren and Rinder [87]. Original FPI devices caused percussive injury using a metal pendulum released from a predetermined height that struck a piston and drove saline into exposed intact dura. Contemporary FPI devices have computer-controlled pneumatic pistons that drive saline into the exposed dura, resulting in improved reproducibility [67]. Lateral [103], medial [102], and repetitive [34] injury models were developed in rats, and the parasagittal FPI injury model was also adapted to mice [17]. Only a few studies exist where FPI was used to study the effects of TBI on AD related pathology. In rats, 6 months after a lateral FPI AD related neuropathology is observed, including immunoreactivity to phosphorylated Tau and Aβ, as well as gradual cortical neuronal loss [55]. In adult rats, FPI injury causes progressive loss of dopaminergic neurons and also enhances vulnerability to Paraquat, a pesticide linked to PD in humans [56]. In this study Paraquat and TBI was the only condition under which amorphous α-syn intracellular aggregates developed suggesting a potential synergistic pathology [56]. Moreover, pioglitazone a drug prescribed to treat diabetes, has been shown to decrease levels of pro inflammatory cytokines, number of activated microglia around dopaminergic axons, and promote dopaminergic neuronal survival [88]. It would be of interest to determine the degree to which transsynaptic spread of α-syn pathology is seen in FPI models that are also exposed to Paraquat. While the importance of FPI to our understanding of the biology that drives TBI pathology cannot be overstated, there are some limitations. FPI models are limited to same-day injuries and require a persistent craniectomy limiting our ability to study chronic effects of repetitive injuries on PD pathology.

#### Penetrating ballistic brain injury (PBBI)

Recent mass shootings in the U.S. have cast a public spotlight on gun-related injuries. In the U.S., 67,000 people are injured by ballistic firearms each year, making ballistic injury from firearms a major health concern [41]. Injuries to the head make up 10% of total gun related injuries [41] and only a third of these injuries are survived [11]. Still, thousands of individuals survive ballistic head trauma each year that predisposes them to neurodegenerative diseases later in life and warrants additional attention. The PBBI model, originally developed using mixed breed cats, consists of right frontal sinus removal thereby exposing the posterior sinus wall. A steel sphere is fired and penetrates the right frontal bone, passing through the entire length of the right cerebral hemisphere [18, 20]. More recently, a PBBI model was developed in rats, and particular ballistic injury parameters were scaled from human to rat proportional to high-velocity North Atlantic Treaty Organization (NATO) round. A PBBI probe was inserted through the right frontal pole of the brain, and a balloon was inflated to 10% of total brain volume [162]. The probe delivered a survivable injury resulting in hemorrhage and necrosis in the nuclei that are also affected in PD (e.g., striatum and substantia nigra) as well as the cortex [162]. PBBI was not used in studies of AD or PD, but it would be of interest to the field to see how the most severe survivable brain injuries that occur in humans, affect susceptibility to development of PD pathology.

#### Weight drop

Weight drop injury models typically consist of a free-falling weight released from a predetermined height down a guide tube striking the animal’s head (shielded or unshielded) or a temporary implant intended to focus the energy to a particular part of the skull. Weight drop models result in either diffuse axonal or focal injuries, the models can be further divided into Marmarou variants that allow for rotational acceleration and the Shoami model where the head of the animal is immobilized. The Marmarou model, developed in 1994, consists of a weight striking an anesthetized rat with a fixed stainless-steel disc preventing skull fracture [96]. The animal is placed on a sponge to allow for rotational acceleration. This model has been widely used to study TBI due to its ability to cause similar type of injury as seen in FPI without the need for a craniotomy. Several variants of the Marmarou model were created in both mice and rats. In the Maryland model, a Marmarou variant, force is applied to the frontal cranium through small metal implants. A temporary brass bar is implanted against the malar process on each side of the rat skull through an infraorbital incision, and these two bars are attached to a cylinder that is struck by a steel ball in free-roll down a ramp [73]. The injury causes anterior-posterior as well as sagittal rotational acceleration. Histological analysis of the brain tissue shows parenchymal bleeding, activated caspase-3 and β-Amyloid precursor protein accumulation in the axons [73]. Another modified Marmarou rat model was developed by Buchele et al. (2016). In their model, the shielded head is struck at a 70-degree angle to accommodate electroencephalography/myography (EEG/EMG) electrodes in order to obtain electrophysiological recordings of vigilance states [16]. Limited space on the rat cranium prevented real time EEG/EMG recordings following a TBI so Buchele et al modified the Marmarou model by moving the impact target 2 mm anterior to bregma over the midline, allowing space on the calvarium for electrode implants. While they find no abnormalities between the sleep/wake cycle of injured and uninjured animals, Buchele et al. report memory deficits and microbleeds in the injured animals [16].

Using a similar setup scaled to mice, Zohar et al. developed a closed head mild TBI model that also does not require preparatory surgery. Anesthetized male ICR mice are placed under a vertical guide tube, through which a weight is dropped [169]. The mice are manually stabilized under the guide tube on a sponge so that temporal right side of the head between the eye and ear are targeted [169]. This closed head injury causes significant and irreversible learning impairments without causing damage to the skin, skull, or a compromised blood brain barrier. Advantages of this model are high survivability, ease of use, and, most importantly, the absence of preparatory surgeries. The same model developed by Zohar et al. (2003) was adopted by Saykally et al. to study how repetitive mild injuries alter protein expression and dendritic complexity in mice. The findings indicate that TDP-43 protein levels increase in the cortex ipsilateral to injury after day 3 and return to normal by 30 and 60 days post-injury, while the TDP-43 expression in the ipsilateral hippocampus increases only after 60 days [133]. They also reported a decrease in relative expression of autophagy factors and decreased dendritic arbor density.

Shohami mouse and rat injury models differ from Marmrou and other similar models in that the head is unshielded, the same impactor (with size-appropriate endpoints) is used for mice and rats, and the head is immobilized. The anesthetized animal is placed on a hard surface, skin is retracted exposing the skull, and the head is immobilized with 4 metal bars. The injury is delivered by a dropped weight [23]. The Shohami injury model produces more profound injuries whilst maintaining an intact skull, resulting in behavioral deficits and large cortical necrotic zones at the levels of hippocampus. While the intent of the Shohami injury device is to cause a focal injury, it is not difficult to imagine that the force of impact is distributed by the intact skull to other brain regions distal from the focal point of injury.

Several Alzheimer’s disease/TBI studies were conducted using weight drop injury models, but only a single PD study has been conducted to date. Using a well-established 6-hydroxydopamine (6-OHDA) model of toxin induced PD together with a closed head weight drop model of injury, De Oliveira showed that a moderate TBI can increase vulnerability to neurotoxicity induced by 6-OHDA [33]. TBI + 6-OHDA caused impaired motor performance on a rotorod that was alleviated with L-DOPA, compromised the BBB, and decreased dopaminergic projections into the striatum [33]. However, in this study like other Parkinson’s disease and TBI studies to date pathology reported lacked phospho-serine 129 positive, skein or corkscrew shaped Lewy neurite-like structures and dense Lewy body-like aggregates typical of human idiopathic PD.

### Future direction: Leucine Rich Repeat Kinase 2 (LRRK2) and Rab proteins

Protein aggregation is a histological hallmark of both PD and TBI and understanding the upstream mechanisms that may bring about this aggregation and subsequent cell death merit further study. Recent focus has been on Leucine rich Repeat Kinas 2, due to its involvement in familial and idiopathic PD and do to its drugability.

The G2019S gain of function mutation in the LRRK2 protein, resulting in hyperactive kinase activity, is the most frequent genetic cause of late onset PD that is pathologically indistinguishable from idiopathic PD [29]. This increase in LRRK2 activity, however, is not found in idiopathic PD. LRRK2 is constitutively expressed in the brain, and the expression levels do not change with age or in idiopathic PD [168]. Furthermore, modest overexpression of LRRK2 alone in animal models has failed to cause selective loss of A9 neurons in the SNpc or produce robust α-syn pathology [78, 84]. A recent hallmark study by Steger et al. (2016) identified a subset of Rab GTPase proteins as exclusive physiological substrates of Leucine-rich repeat kinase 2 (LRRK2) [144]. The study also shows that the hyperactive LRRK2 mutation shifts subcellular distribution of Rabs from cytosolic to membrane bound, which may adversely impact homeostatic Rab function [144]. Rab32, Rab11, Rab5, and Rab7 have been shown to operate in mitochondrial function, dopamine transport, and late endosome-lysosome formation, respectively, all processes which are disrupted in PD. Furthermore, these and other Rabs have also been implicated in PD prior to identification of Rabs as physiological LRRK2 substrates. LRRK2 and Rab interactions may normally be controlled by spatial and temporal expression of Rabs that are disrupted by TBI. It is therefore possible that in the absence of PD-inducing LRRK2 mutations, TBI may play a major role. As the most common non-genetic risk factor for PD, mTBI may be responsible for increasing LRRK2 substrate availability by chronically elevating the expression levels of Rabs in the brain, causing selective loss of susceptible neurons. During an infection Rab proteins are rapidly mobilized in immune cells suggesting that they are responsive to external aversive stimuli [119]. What remains to be seen is if TBI increases expression of Rabs thereby providing abnormal substrates for LRRK2 in neurons and glial cells which may cause TBI induced PD.

## Conclusion

TBI is a significant non-genetic risk factor for developing PD later in life. While TBI has been implicated in several neurodegenerative disorders the strongest emerging evidence is for a causative relationship with late onset PD. Accumulating evidence suggests that inflammation may play a significant role in PD pathogenesis following TBI, therefore animal models that do not require preparatory surgeries should be considered to avoid confounding results of injury. According to current literature, there is a significant pathological overlap between late onset PD and TBI including protein aggregation, but the area of research in animal models remains under-explored with only a few animal studies focusing on specific aspects of PD pathology using TBI models. Better understanding of the mechanisms prior to the onset of neurodegeneration, like the interactions between LRRK2 and Rabs may uncover targets amenable to pharmacological intervention which could lead to preventative treatments for PD following a TBI.

## Data Availability

NA
